# Intergovernmental Learning Exchange to Advance Data-Driven Decision Making: Experiences from Nigeria.

**DOI:** 10.12688/f1000research.166773.2

**Published:** 2026-02-23

**Authors:** Agbonkhese I. Oaiya, Oludare Onimode, Olaposi Olatoregun, Gibril Gomez, Bashorun Adebobola, Christopher Isokpunwu, Oladejo Folasade, Balogun Olufunke, Herman Tolentino, Brianna Musselman, Sam Wambugu

**Affiliations:** 1 PATH, FCT Abuja, FCT Abuja, Nigeria; 2 APIN Public Health Initiative/Public Health Information Surveillance, Systems and Solutions (PHIS3), FCT Abuja, FCT Abuja, Nigeria; 3 National HIV/AIDS, Hepatitis and STIs Control Programme (NASCP), Federal Ministry of Health and Social Welfare, FCT Abuja, FCT Abuja, Nigeria; 4 Health Planning, Research and Statistics, Federal Ministry of Health and Social Welfare, FCT Abuja, FCT Abuja, Nigeria; 5 Federal Ministry of Women Affairs, FCT Abuja, FCT Abuja, Nigeria; 6 Nigeria Ministry of Defence, FCT Abuja, FCT Abuja, Nigeria; 7 University of Illinois Chicago, Chicago, Illinois, USA; 8 PATH, Seattle, USA

**Keywords:** Intergovernmental Learning Exchange to Advance Data-Driven Decision Making (I-LEAD), Growing Expertise in E-Health Knowledge and Skills (GEEKS), Public Health Informatics, Digital Health Strategy, Digital Health Governance, Health Information System (HIS), Data-Driven Decision Making, Informatics-Savvy Health Organization (ISHO), Electronic Medical Records (EMR), National Data Repository (NDR), Interoperability, Capacity Building, Leadership Development, HHIV/AIDS Programs, Low- and Middle-Income Countries (LMICs), Nigeria

## Abstract

**Background:**

Nigeria has strategically invested in digital health to achieve HIV/AIDS epidemic control, meet SDG health targets, and advance towards UHC. Despite progress, challenges persist. This paper details Nigeria’s commitment, in collaboration with PEPFAR, CDC, and other agencies, to address Health Information System (HIS) challenges through participation in the Intergovernmental Learning Exchange to Advance Data-Driven Decision Making (I-LEAD) programme.

**Methods:**

The I-LEAD programme followed a three-phase approach: 1) conducted an expedited Informatics-Savvy Health Organisation (ISHO) assessment to identify critical national HIS challenges; 2) enhanced informatics capabilities of selected Nigerian delegates, including a purpose-fit session, Bring Your Own Difficult Decision (BYODD), involving SMEs to collaboratively refine, contextualise and guide the localised development of actionable solutions for national HIS challenges; and 3) outlined the nation’s approach to implementing the HIS solutions

**Results:**

The expedited ISHO assessment identified five HIS challenges: governance, interoperability, data security, Electronic Medical Record (EMR) centralisation, and funding. Participating in the I-LEAD programme strengthened Nigeria’s leadership technical capacity in informatics, particularly in strategic visioning and planning, with the BYODD sessions resulting in the collaborative development of localised solutions to address the five HIS challenges. In the post-I-LEAD phase, efforts focused on two of the HIS solutions. These activities are 1) improving data quality through harmonisation of value data sets, and 2) decentralising I-LEAD learning and building the capacity of Public Health Informatics (PHI) technical groups through progressive levels of Growing Expertise in E-Health Knowledge and Skills (GEEKS) training. These activities were selected because of their potential to deliver the maximum impact within the HIS ecosystem.

**Conclusion:**

Nigeria’s active participation and commitment through the I-LEAD programme have strengthened its digital health agenda, leveraging health informatics to enhance healthcare delivery and achieve broader health goals. This approach can serve as a model for other developing nations facing similar health informatics hurdles.

## Introduction

Globally, the widespread integration of digital technology has transformed how various industries operate, including those in the healthcare sector. Digital technology in health increases the accessibility to medical information, telemedicine, electronic health records, and cutting-edge diagnostics, resulting in more efficient, personalised, accessible, and improved delivery of quality healthcare services. ^1^ The widespread adoption of these technologies in health is expected to increase the potential for global connectedness by growing countries’ progress towards bridging the digital divide within their respective health systems, developing informatics-savvy groups to secure and sustain these interoperable systems, and achieving improved quality of healthcare for all. ^1, 2^ Nigeria is one such developing country that has made significant investments in the digital health space to bridge the digital divide to achieve Human Immunodeficiency Virus and Acquired Immune Deficiency Syndrome (HIV/AIDS) epidemic control and achieve its vision to be a nation free from the Acquired Immune Deficiency Syndrome (AIDS) disease, meet the 2030 agenda by accelerating progress across the health-related Sustainable Development Goals (SDG), and ultimately, advance towards Universal Health Coverage (UHC) for her populace. ^3^

Nigeria’s digital health sector has witnessed remarkable growth and transformation in recent years. The country has recognised the pivotal role of digital technology in improving healthcare access and delivery, especially in the diagnosis, treatment and management of HIV/AIDS disease, which is a known contributor to national mortality rates. Presently, the Nigerian digital health ecosystem comprises several global health technologies that have been adapted and implemented to enhance patient care and streamline healthcare management. ^3– 6^ These technologies include Health Information Systems (HIS) like the National Health Management Information System (NHMIS) driven by the District Health Information System (DHIS2), the National Health Workforce Registry (NHWR) that is based on the iHRIS Manage Software, Electronic Medical Records (EMR) based on OpenMRS and other platforms, and a National Data Repository (NDR), leveraging popular Free and Opensource solutions. ^3– 7^ In addition, the government of Nigeria, in partnership with global organisations like the United States’ President’s Emergency Plan for AIDS Relief (PEPFAR), has also initiated numerous programmes aimed at optimising digital healthcare governance and enhancing the infrastructure base, and there has been remarkable achievement. ^8– 10^ The country has also adopted a national eHealth strategy and policy framework to oversee the incorporation of technology into the healthcare sector, encouraging the seamless exchange of data among healthcare institutions. However, some challenges persist.

Digital health governance in Nigeria is not fully functional when compared to the global benchmark. ^6^ While there is an eHealth strategy in place, complete with a monitoring and evaluation framework, the strategy faces challenges when it comes to practical implementation. Various digital technological solutions have been developed independently, often without adherence to the recommended health information standards for data exchange, transmission, and messaging, as well as security, privacy, and hardware. These siloed and isolated initiatives limit the smooth exchange of data among them, consequently affecting the quality of healthcare services rendered at the various points of care and impeding collaborative efforts in reducing the epidemic impact of HIV/AIDS. Additionally, there are concerns regarding financing digital health in the country. ^6^ Finding a sustainable funding model remains a challenge for many projects and programs, and, notably, the absence of a workforce proficient in informatics to effectively plan for, manage and sustain the digital health sector further complicates the landscape.

This paper outlines the measures taken by the Government of Nigeria, spearheaded by the Federal Ministry of Health and Social Welfare (FMoH&SW) through the National AIDS, Viral Hepatitis and STI Control Programme (NASCP), along with other ministries such as the Federal Ministry of Women Affairs (FMoWA) and the Nigerian Ministry of Defence (NMoD) and in collaboration with key international partners. These efforts were supported by the United States’ President’s Emergency Plan for AIDS Relief (PEPFAR) and its implementing agencies, including the Centres for Disease Control and Prevention (CDC), the Department of Defense (DoD), and the United States Agency for International Development (USAID). Together, these diverse stakeholders worked to identify and address common national HIS challenges, with a focus on optimising and operationalising the national digital health governance, enhancing the coordination of HIV/AIDS disease programme areas to attain epidemic control, and, more importantly, cultivating an informatics-savvy workforce able to sustain the Nigerian digital health ecosystem. These were achieved through participation and commitment to the Intergovernmental Learning Exchange to Advance Data-Driven Decision Making (I-LEAD) programme.

The I-LEAD programme aims to empower leaders, managers, and technical experts engaged in Public Health Informatics (PHI) in envisioning the digital health landscape, formulating effective strategies to realise their vision, and successfully implementing and sustaining these strategies. This becomes especially valuable for Nigeria, as the nation endeavours to harness digital investments and solutions to enhance healthcare delivery, achieve a nation free of AIDS, and ultimately attain UHC for its population.

## Methods

### Setting the context

It has been nearly four decades since the outbreak of Nigeria’s first HIV/AIDS case. Since then, the government of Nigeria, with support from international partners such as PEPFAR, has been actively involved in the fight against HIV/AIDS to attain epidemic control and achieve an AIDS-free Nigeria, with zero new infections, zero discrimination and stigma and zero AIDS-related deaths. ^11^ Recently, the Nigerian government has taken significant steps towards optimising the management of patient health information, elevating data quality, and fostering a more efficient and coordinated approach to healthcare services, particularly within the realm of HIV/AIDS programme management. To support this paradigm shift, PEPFAR, through its agency partners; CDC, USAID and DoD, and in-country technical partner, APIN Public Health Initiative/Public Health Information Surveillance, Systems and Solutions (APIN/PHIS3), partnered with the government of Nigeria in institutionalising relevant eHealth technologies in alignment with the recommended National Health Information Architecture. As of December 2023, EMRs have been deployed across a total of 2,439 health facilities in all states in Nigeria, including the Federal Capital Territory, Abuja. In addition, a central repository of de-identified patient-level data aggregated from these service delivery points, called the National Data Repository (NDR), has also been deployed with over 1.9 million patients actively in treatment as of the same date. ^12^ Apart from implementing these eHealth technologies, a series of multifaceted and phased capacity-building sessions was also conducted to drive ownership and sustainability post-donor support.

Some of these learning and development workshops were designed to align with the various administrative structures of the government, emphasising broad health informatics-related activities such as strategy development. Other sessions delved into the practical implementation of these strategies by building capacity for the relevant actions and procedures needed to translate these strategic goals into actionable activities. Additionally, some workshops focused on the effective utilisation of institutionalised digital tools within the context of HIV/AIDS care. Nevertheless, challenges were encountered in establishing the crucial link between formulating a robust national digital health vision, creating comprehensive cross-cutting strategies that spanned all disease and health areas, and effectively translating these long-term and medium-term strategies into operational actions to foster the growth of the digital landscape in Nigeria.

### Intergovernmental Learning Exchange to Advance Data-Driven Decision Making (I-LEAD)

The Intergovernmental Learning Exchange to Advance Data-Driven Decision Making, which is commonly referred to as I-LEAD, is a programme that aims to empower diverse country-level leaders beyond decision-making and policy formulation capacities, managers and technical experts in the public health informatics space. The programme supports these nationally diverse stakeholders in envisioning the digital health landscape, formulating effective strategies to realise their vision, and successfully implementing and sustaining these strategies for impact.

To ensure effective planning, implementation, and sustainability of national digital health solutions and their impact on ensuring the delivery of care, PHI professionals in leadership and managerial capacities should cultivate proficiency across four fundamental dimensions: Vision, Strategy, Operations, and Tactics (VSOT). ^13^ TThe VSOT framework was not developed as part of this study; rather, the framework represents a core PHI leadership construct underpinning the design and delivery of the I-LEAD programme, and another complementing programme called Growing Expertise in E-Health Knowledge and Skills (GEEKS).

The I-LEAD programme primarily focuses on strengthening competencies related to vision and strategy, while completion of I-LEAD is complemented by the GEEKS programme, which addresses the remaining two dimensions: operations and tactics. Together, the synergy of combining these learning programmes, I-LEAD and GEEKS, results in a collective strengthening of the proficiency of a diverse spectrum of PHI stakeholders. The combined approach, which addresses all four VSOT framework dimensions, guarantees that national entities, including PHI leaders and decision-makers or policy formulators, managers, and technical experts, who are essential in conceiving the digital health landscape and developing strategies to realise it, are better prepared to realise their vision, and effectively execute and maintain these strategies.

The I-LEAD programme is conducted on a per-demand basis through the PEPFAR partner – CDC headquarters, and the frequency depends on the requests received from countries that are expected to commit to the programme requirements. One crucial commitment involves following through with the roadmap as an integral part of the post I-LEAD phase. Nigeria, through the FMoH&SW/NASCP and its PEPFAR partners, expressed their interest in attending the 2023 edition, which also included representation from other countries in the eastern African region.

### Establishing a core technical team

To ensure Nigeria’s active involvement in the I-LEAD programme, a core technical team was established, comprising representatives from the FMoH&SW/NASCP, APIN/PHIS3, and the Technical Assistance Platform (TAP) - a CDC-supported project driven by PATH that aims to assist Nigeria in harnessing and implementing sustainable digital health technologies. This core team had two primary responsibilities, with the first securing Nigeria’s participation in the I-LEAD event. The core team collaborated with the I-LEAD organisers, notably staff from the CDC headquarters in Atlanta, to provide orientation on the prerequisites and processes for attending the I-LEAD event. This orientation also covered Nigeria’s commitment post I-LEAD. The second core team’s responsibility was to coordinate the Nigerian delegates attending the event in completing the I-LEAD preparatory activities. One such activity was leveraging an evidence-based method, the Informatics-Savvy Health Organisation (ISHO) assessment framework, to identify national HIS challenges to bring to the event.

### Formulating the Nigerian delegates to participate in the I-LEAD

The technical core team purposefully identified and selected 17 delegates to represent Nigeria in the I-LEAD programme. The number of delegates was informed by the scope and structure of the HIV/AIDS programme in Nigeria, and the need to ensure a balanced representation across key institutions involved in national HIS governance, planning and implementation. The Nigerian delegation included government representatives from the FMoH&SW/NASCP, FMoWA, and NMoD, as well as individuals from various inter-agency organisations such as the CDC, DoD, USAID, APIN/PHIS3, Palladium Group/Data.Fi, and PATH.

With support from the I-LEAD organisers and the core technical team, the delegates underwent a comprehensive orientation that covered various aspects of the I-LEAD process in optimising country HIS ecosystems. This included a detailed explanation of the prerequisites for participation and a thorough understanding of Nigeria’s commitment to the post I-LEAD activities. In addition, the delegates were trained to enhance their capacities in utilising the ISHO framework in assessing national informatics systems readiness, identifying HIS gaps across key health system domains impacting healthcare service delivery, and using those insights to inform strategic planning activities and health system strengthening initiatives. Notably, identifying these national HIS gaps must be done empirically through the conduct of an ISHO assessment. This is a fundamental requirement for participating in the I-LEAD programme.

### Conducting an ISHO assessment

ISHO stands for Informatics-Savvy Health Organisation. ^14, 15^ The ISHO toolkit was originally developed by the Public Health Informatics Institute (PHII) to guide governments, agencies, or any organisation in assessing their informatics readiness and capabilities, to develop e-Health strategies, information systems, electronic health information exchanges, and ensure the availability of an informatics-savvy workforce to achieve a digital public health future. ^14^ The ISHO toolkits consist of two types of tools: above-site and site-level. Both tools achieve their goal by assessing informatics capabilities across three strategic pillars. The first pillar, “Vision, Policy, and Governance”, focuses on the existing digital health governing structures, delving into the implementation status of current digital health policies, strategies, and costed plans. The second is “Skilled Workforce”, which examines the structures that ensure the continuous supply and availability of informatics-savvy health workers. The last pillar is “Effective Information Systems”, which evaluates the technological aspects of the current information technology capabilities. ^15– 21^

Using the above-site ISHO tool, the Nigerian delegates conducted an expedited ISHO assessment at the national level in Nigeria. Each delegate, representing key government ministries including the FMoH&SW and NASCP, the FMoWA, the NMoD, and implementing partners supporting the Nigerian government HIV/AIDS response, played a key role in conducting the assessment. The goal of the evaluation was to determine national HIS strengths, as well as identify informatics challenges, towards establishing a national baseline. Furthermore, completing this stage is a prerequisite for taking part in a Bring Your Own Difficult Decision (BYODD) session during the I-LEAD event. This interactive session provides the platform where critical HIS challenges in participating countries are collectively addressed with support from global public health informatics experts.

The completed above-site ISHO tools were collated, after which the Nigerian delegates convened multiple times to collectively review and analyse each completed above-site tool. Responses from each completed above-site tool were aggregated in a structured format to identify recurring patterns across the different evaluated domains, synthesise emergent themes and collectively shortlist the HIS challenges for presentation in the I-LEAD’s BYODD session.

### Prioritising national HIS gaps

The national HIS gaps identified after conducting the expedited above-site ISHO assessment were further shortlisted. This was done after several consultative meetings with the government of Nigeria and health informatics Subject Matter Experts (SME) internally and externally, from the CDC headquarters. A selection criterion was developed, and factors such as implementation impact in the digital health space, required resources such as Human Resources for Health (HRH) and cost of implementation, as well as the anticipated timelines, were used to rank and prioritise HIS challenges.

## Results

### ISHO assessment findings

Several health informatics challenges were identified following the analysis of the expedited above-site ISHO assessment and presented in Figure 2. Issues around HIS governance and coordination were a notable challenge. Others revolved around data security, systems interoperability, EMR centralisation and strategies for improvement of data quality, data management and use, sustainability, and funding.

Further discussions were held amongst the delegates attending the I-LEAD and health informatics SME from the government of Nigeria and PEPFAR to distil the identified gaps into five that would be addressed at the I-LEAD event. The foremost informatics gap centred on (1) governance, while others revolved around (2) information system interoperability, (3) data security, (4) EMR centralisation to address the availability of 3 fully established EMRs in Nigeria, and (5) securing digital health funding.

### Outputs from I-LEAD’s BYODD sessions

The Nigerian delegate’s capacity was built across various aspects in envisioning, conceptualising, and developing country-level informatics strategies using multiple evidence-based problem-solving methodologies. With respect to the BYODD sessions, these comprised numerous iterative sessions with SMEs. During these sessions, HIS challenges specific to Nigeria were presented and rigorously examined, drawing on different problem-solving methodologies and other country experiences, all tailored to the unique local context. The Nigerian HIS challenges were subsequently addressed and redefined, with five proposed solutions presented in Table 1. Of the five proposed solutions, four were HIS governance-related. These were proposed due to the peculiarities of our multi-stakeholder environment, ensuring coordination and focused intervention to achieve considerable impact with modest resource requirements through efficient implementation planning.

**Table 1.** Redefined HIS challenges after BYODD sessions.

HIS challenge domain	Activity	Description	Priority
*HIS Governance and Systems Interoperability	Improving data quality through harmonisation of value data sets	Improve data exchange between all EMRs in Nigeria, NigeriaMRS, LAMISPlus and ViVa, with the NDR through harmonisation of value data sets	* **High (5)**
Knowledge Sharing and Sustainability	Decentralise I-LEAD learning and/or build the capacity of PHI technical groups by convening a TIER 1 and 2 GEEKS training	This activity focuses on harnessing the benefits of the VSOT framework by combining the I-LEAD and GEEKS programmes. This activity shall foremost decentralise the knowledge gained during the I-LEAD and then plan for and conduct a GEEKS programme for technical experts	* **High (5)**
HIS Governance	Digital Health Strategy Implementation and Use	Operationalise and monitor the implementation of the revised Nigeria Digital Health Strategy to enhance the effectiveness of streamlined healthcare operations	High (4)
HIS Governance	Addressing digital health data standards challenges through TA for the Digital Health Standards Subcommittee	Technical assistance (TA) to support digital health standards subcommittees of the National Digital Health Technical Committee (NDHTC)	High (4)
HIS Governance (Security)	Conduct security vulnerability and risk assessment on EMR and NDR systems	Identifying and addressing potential security vulnerabilities and risks within the EMR and NDR	Medium (3)

Abbreviations: EMR, Electronic Medical Records; GEEKS, Growing Expertise in E-Health Knowledge and Skills; HIS, Health Information System; I-LEAD, Intergovernmental Learning Exchange to Advance Data-Driven Decision Making; NDR, National Data Repository; PHI, Public Health Informatics; TA, Technical Assistance; NDHTC, National Digital Health Technical Committee; VSOT, Vision, Strategy, Operations, and Tactics.

* Activity selected for implementation by the Nigerian delegates as part of the post I-LEAD phase.

The first HIS governance streamlined activity focused on improving data quality through the harmonisation of value data sets across all EMRs in the country.

The second activity identified from the broader HIS governance challenges focused on the existing Nigerian digital health strategy, specifically, on its implementation and role in supporting interoperability across key information systems to optimise healthcare service delivery. These included activities related to operationalising and monitoring the rollout of the revised strategy. Addressing digital health data standards challenges through technical assistance for the Nigerian digital health standards subcommittee and conducting a security vulnerability and risk assessment on health information systems involved in HIV/AIDS, EMRs, and NDR systems were the third and fourth streamlined activities. The fifth and last activity was on knowledge transfer to promote ownership and sustainability post-donor support. This focused on decentralising the knowledge gained from the I-LEAD, harnessing the fullness of the VSOT framework by conducting a progressive GEEKS event. A detailed roadmap outlining the various tasks across the streamlined activities presented in Table 1 was developed and is provided in Appendix I.

Finally, as part of the Nigerian government’s commitment to the I-LEAD programme, two activities were prioritised for implementation. These included improving data quality through harmonisation of value data sets and decentralising I-LEAD learning and building the capacity of PHI technical groups by convening a tier 1 and 2 GEEKS training.

## Discussion

In preparation for the I-LEAD programme, the Nigerian delegates engaged in a series of activities to identify common national HIS gaps. These national HIS gaps were identified through an evidence-based approach by conducting an expedited above-site ISHO assessment. Following the assessment, regular, structured consultative meetings and discussion sessions were held. These sessions formed part of routine coordination to review progress and ensure alignment with agreed objectives. These engagements informed the prioritisation of HIS gaps identified through the expedited ISHO assessment, resulting in the selection of five HIS challenges for the I-LEAD event. These meetings were conducted in consultation with the Nigerian technical team and delegates, with support from PHI SMEs in-country and externally from supporting donors and implementing partners.

Participating in the I-LEAD demonstrated successful resolution of the shortlisted HIS gaps. Five practicable strategies with the most significant potential to address the identified national HIS issues were generated based on criteria that were unique to our locality, and these were presented in Table 1 above. Furthermore, two (2) of these strategies were selected and earmarked for implementation by the Nigerian delegates as part of the post I-LEAD commitment. These activities were selected based on criteria such as impact on the digital health space, availability of existing structures or some level of previous work to build on, and the resources required. The first activity cuts across two domains of the Nigerian digital health space: governance and system interoperability. Its objective is to enhance data exchange among all EMRs in Nigeria: NigeriaMRS, LAMISPlus and ViVA EMR, with the NDR through harmonisation of value data sets. The second activity focused on knowledge dissemination and harnessing the benefits of the VSOT framework through participating in the I-LEAD programme. This initiative sought to decentralise I-LEAD learnings, specifically focusing on the first two components of the VSOT framework: vision and strategy. Additionally, it is also aimed to enhance the capabilities of the internal PHI technical groups by organising tier 1 and 2 GEEKS training, with the GEEKS training specifically addressing the last two components of the VSOT framework: operation and tactics.

**Figure 1.**
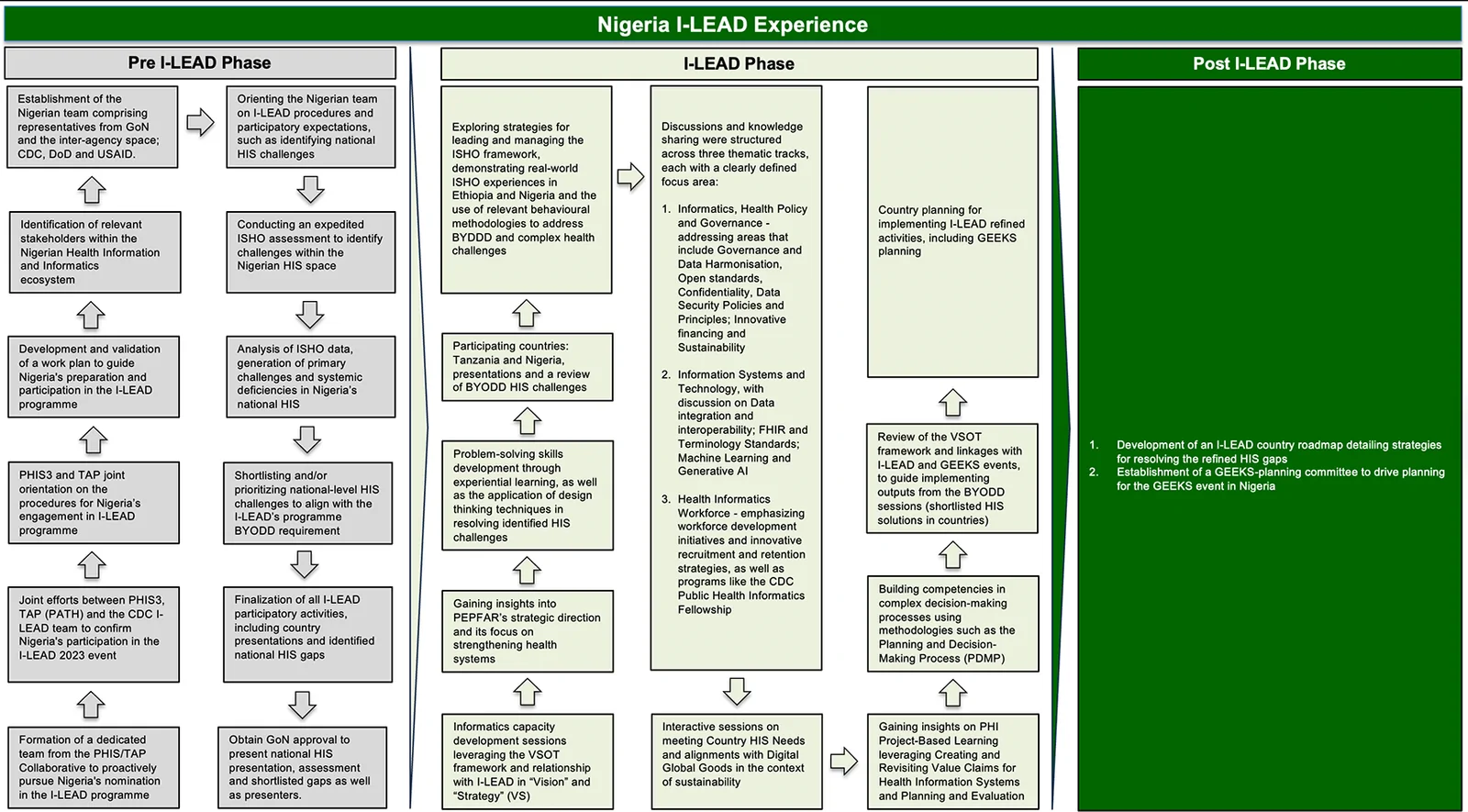
Overview of the Nigeria I-LEAD experience across 3 phases: preparatory activities, the I-LEAD main event, and post I-LEAD activities. Abbreviations: AI, Artificial intelligence; BYODD, Bring Your Own Difficult Decision; CDC, US Centers for Disease Control and Prevention; DoD, United States Department of Defense; FHIR, Fast Healthcare Interoperability Resources; GEEKS, Growing Expertise in E-Health Knowledge and Skills; GoN, Government of Nigeria; HIS, Health information system; I-LEAD, Intergovernmental Learning Exchange to Advance Data-Driven Decision Making; ISHO, Informatics-Savvy Health Organization; PEPFAR, United States President’s Emergency Plan for AIDS Relief; PHIS3, Public Health Information Surveillance, Systems and Solutions; USAID, United States Agency for International Development; TAP, Technical Assistance Platform; VSOT, Vision, Strategy, Operations, and Tactics.

There was a limitation in the I-LEAD approach presented in Figure 1. The limitation was the expedited above-site ISHO assessment conducted to identify and shortlist HIS gaps taken to the I-LEAD event. The evaluation was a rapid one and limited in scope. Although the technical core team embarked on multiple consultative meetings with PHI SMEs locally and externally to mitigate this, we recommend conducting a more comprehensive ISHO assessment, one that encompasses both above-site and site-level evaluations. This expanded assessment would provide a more thorough understanding of HIS challenges and better inform strategies for future improvements.

**Figure 2.**
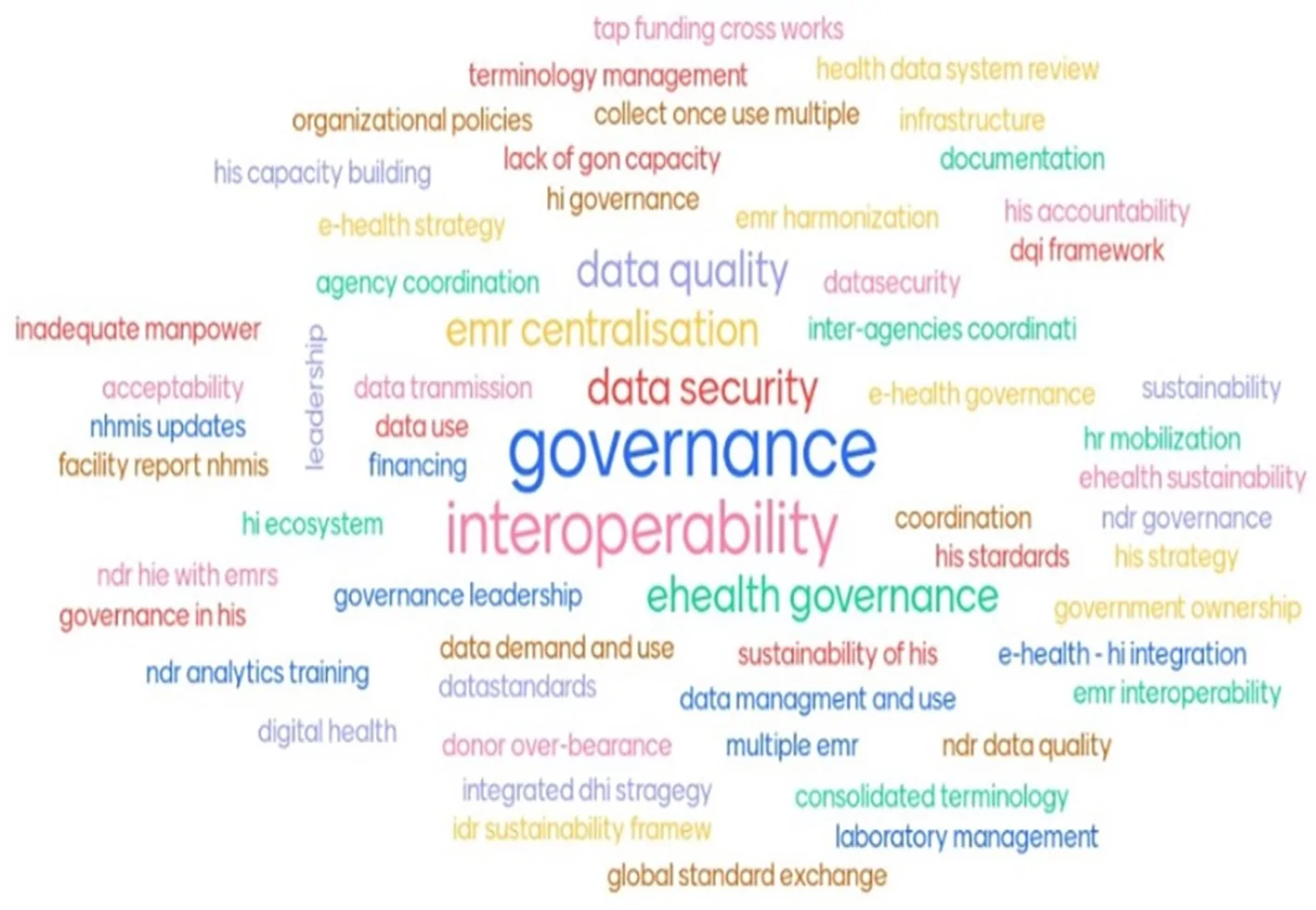
National HIS challenges identified after the expedited above-site ISHO assessment.

## Conclusion

In conclusion, I-LEAD adopts an evidence-based methodology for shaping policies to tackle health informatics challenges countries face. This approach differs significantly from the conventional approach most developing countries use, which is often ineffective and lacks widespread impact in the broader health sector. The I-LEAD approach employs an empirical strategy in determining national health informatics-related challenges. It incorporates a series of capacity-building activities to reinforce PHI’s leadership and management skills. This approach helps in envisioning long-term informatics-related objectives and provides tailored approaches to address challenges to the specific needs of the locality.

For the Nigerian delegation led by the FMoH&SW, participation in the I-LEAD programme resulted in numerous significant advantages. This initiative served as a valuable platform for advancing PHI leadership proficiency in health policy and governance, focusing particularly on key areas such as data sharing, data security, innovative financing, and sustainability. The programme also addressed essential knowledge domains like data integration, interoperability, FHIR and Terminology Standards, as well as Machine Learning and Generative Artificial Intelligence. Additionally, the I-LEAD programme encompassed strategies for enhancing workforce recruitment, development, and retention within the informatics realm. Furthermore, the I-LEAD programme facilitated direct interaction with experts in the health informatics field, enabling the delegation to harness evidence-based techniques for addressing identified PHI challenges in Nigeria. The PHI solutions articulated through this process embedded localisation and sustainability by aligning activities with existing national digital health structures and needs, prioritising high-impact interventions that could be delivered within available resources, and strengthening local capacity through decentralised learning and the GEEKS programme. The FMoH&SW’s commitment to implementing the refined PHI solution was reinforced through their participation in the programme, establishing the groundwork for bridging the digital divide in the country.

Key lessons from the Nigerian experience underscore the value of coupling PHI leadership development with technical skills strengthening, embedded in creating a vision and strategy that is localised, shared, integrated, and aligned across all stakeholders, ahead of implementation. These insights offer practical guidance for other countries seeking to advance their digital health ecosystems through similar intergovernmental learning and capacity-building approaches.

### Implications for policy & practice

The findings and interventions outlined in this report bear significant implications for policy and practice in Nigeria’s healthcare landscape. Firstly, the identification of national health informatics challenges, particularly in governance, interoperability, data security, EMR centralisation, and funding, emphasised the necessity for a thorough and well-coordinated digital health strategy. This will guide policymakers to prioritise establishing, implementing, and monitoring robust governance frameworks to facilitate the smooth integration of various digital health initiatives in Nigeria.

Furthermore, the commitment to address these challenges through the I-LEAD programme signifies a shift towards evidence-based policymaking. The I-LEAD programme incorporated BYODD sessions, which harnessed the expertise of global PHI experts to refine and streamline Nigeria’s HIS challenges into actionable activities with the broadest impact across multiple health domains.

Finally, the emphasis on improving data exchange between EMRs as part of the post I-LEAD phase demonstrates the Nigerian government’s commitment to building a skilled workforce and ensuring the integrity and confidentiality of health data. This phase opens the path for better data-driven decision-making and more efficient healthcare operations. Policymakers can utilise this model to shape future initiatives, creating a robust and responsive digital health ecosystem and acting as a model for other countries facing similar difficulties.

## Disclaimer

The views and opinions expressed in this article are those of the authors and do not necessarily reflect the official policy or position of any affiliated agency of the authors.

## Data Availability

All data supporting the results are included within this article and its related files. Figshare: I-LEAD Experience in Nigeria: Supplementary Data: https://doi.org/10.6084/m9.figshare.29345027. ^22^ This project contains the following data:
•
Figure 1. jpg - Overview of the Nigeria I-LEAD Experience across 3 phases: preparatory activities, the I-LEAD main event, and post I-LEAD activities. •
Figure 2. jpg - National HIS Challenges Identified After Expedited Above-Site ISHO Assessment. •
Table 1 – Redefined HIS Challenges After BYODD Sessions. • Appendix 1 - Nigeria I-LEAD Roadmap.xlsx. • ISHO Expedited Assessment Templates and Completed Tools. Data are available under the terms of the Creative Commons Attribution 4.0 International license (CC-BY 4.0).
