# Supplementary material for: Intergovernmental Learning Exchange to Advance Data-Driven Decision Making: Experiences from Nigeria
Source: F1000Res. 2026 Feb 23;14:759. Originally published 2025 Aug 1. [Version 2] doi: 10.12688/f1000research.166773.2 (PMC12933044; doi:10.12688/f1000research.166773.2)
Supplement: Peer review history

# Reviewer report: Reviewer response for version 2

**DOI:** 10.5256/f1000research.196534.r412147

**Published:** 2026-08-17

**Authors:** Omid Panahi

**Affiliations:** 1 Centro Escolar University, Manila, Metro Manila, Philippines

This manuscript presents a relevant case study of Nigeria’s participation in the Intergovernmental Learning Exchange to Advance Data-Driven Decision Making (I-LEAD) programme. The authors describe a structured three-phase approach involving an expedited Informatics-Savvy Health Organisation (ISHO) assessment, capacity building and problem-solving through the I-LEAD/BYODD process, and subsequent post-I-LEAD implementation planning.

 The study identifies five major health information system challenges in Nigeria: governance, interoperability, data security, EMR centralisation, and funding. It subsequently describes five proposed strategies and the prioritisation of two interventions focusing on harmonisation of value data sets and decentralisation of I-LEAD learning through GEEKS training.

 Overall, the revised version is clearly presented and provides useful practical information regarding digital-health governance, public health informatics capacity building, interoperability, and sustainability in an LMIC setting.

 1. Presentation and current literature — **Yes**

 The manuscript is clearly organised and provides appropriate background on Nigeria's digital-health ecosystem, including health information systems, EMRs, the National Data Repository, digital-health governance, and workforce capacity.

 The references include relevant WHO, Nigerian government, PEPFAR, OpenMRS, and health-informatics literature. The revised manuscript also appropriately clarifies the origin of the VSOT framework and distinguishes it from the authors' own programme activities.

 2. Study design and technical soundness — **Yes**

 For a programme-based case study describing the Nigerian I-LEAD experience, the methodological approach is appropriate. The use of the ISHO assessment followed by structured consultations, prioritisation of challenges, BYODD sessions, and development of locally adapted solutions provides a coherent framework for the objectives of the article.

 The authors have also appropriately acknowledged the limitation of using an expedited above-site ISHO assessment and recommend a more comprehensive assessment incorporating site-level evaluation.

 3. Methods and analysis/reproducibility — **Yes**

 The revised manuscript provides sufficient information to understand how the 17 delegates were selected, how the expedited ISHO assessment was conducted, how findings were aggregated and prioritised, and how the BYODD process contributed to the development of proposed solutions.

 Importantly, the underlying assessment materials, figures, table, and implementation roadmap are made available through the supplementary data repository. This substantially strengthens reproducibility.

 A minor opportunity for improvement would be to provide more quantitative information regarding the prioritisation process or, if available, objective measures of changes in participant competencies. However, these are not essential to establish the scientific validity of the current case study.

 4. Statistical analysis — **Not applicable**

 The manuscript does not present inferential statistical analyses. The work primarily reports a programme experience, qualitative/structured assessment process, prioritisation activities, and implementation planning. Therefore, formal statistical analysis is not applicable.

 5. Source data availability — **Yes**

 The authors provide supplementary materials through Figshare, including the ISHO assessment templates and completed tools, Figure 1, Figure 2, Table 1, and the implementation roadmap. This provides appropriate supporting documentation for the reported findings and enhances transparency and reproducibility.

 6. Conclusions — **Yes**

 The conclusions are generally supported by the findings presented. The authors appropriately describe the benefits of the I-LEAD experience in strengthening public health informatics leadership, strategic planning, stakeholder coordination, and locally adapted solutions.

 The manuscript does not appear to claim that the programme has definitively improved population-level health outcomes. Instead, it describes the programme's contribution to capacity building and the development of actionable HIS strategies, which is consistent with the evidence presented.

 The acknowledgement of the limitations of the expedited ISHO assessment is also appropriate.

 Overall assessment

 The revised manuscript is scientifically sound for its intended purpose as a programme-based case study and provides useful practical lessons for strengthening digital-health and health-information-system capacity in Nigeria and potentially other LMICs.

**No mandatory scientific revisions are required.** Minor editorial improvements and, if available, inclusion of objective measures of programme impact could further strengthen the manuscript, but these are not necessary for scientific validity.

**Overall recommendation: Approved with Reservations.**

Is the work clearly and accurately presented and does it cite the current literature?

Yes

If applicable, is the statistical analysis and its interpretation appropriate?

Not applicable

Are all the source data underlying the results available to ensure full reproducibility?

Yes

Is the study design appropriate and is the work technically sound?

Yes

Are the conclusions drawn adequately supported by the results?

Yes

Are sufficient details of methods and analysis provided to allow replication by others?

Yes

Reviewer Expertise:

Digital Health; Health Informatics; Artificial Intelligence in Healthcare; Public Health Informatics; Health Information Systems; Healthcare Data Analytics; Machine Learning Applications in Healthcare; Digital Health Governance; Interoperability and Electronic Health Records; Global Health.

I confirm that I have read this submission and believe that I have an appropriate level of expertise to confirm that it is of an acceptable scientific standard, however I have significant reservations, as outlined above.

# Reviewer report: Reviewer response for version 2

**DOI:** 10.5256/f1000research.196534.r461432

**Published:** 2026-02-24

**Authors:** Rok Hrzic

**Affiliations:** 1 Maastricht University, Maastricht, The Netherlands

The authors sufficiently addressed my comments.

Is the work clearly and accurately presented and does it cite the current literature?

Partly

If applicable, is the statistical analysis and its interpretation appropriate?

Not applicable

Are all the source data underlying the results available to ensure full reproducibility?

No

Is the study design appropriate and is the work technically sound?

Partly

Are the conclusions drawn adequately supported by the results?

Partly

Are sufficient details of methods and analysis provided to allow replication by others?

No

Reviewer Expertise:

comparative health (policy) research, workforce development

I confirm that I have read this submission and believe that I have an appropriate level of expertise to confirm that it is of an acceptable scientific standard.

# Reviewer report: Reviewer response for version 1

**DOI:** 10.5256/f1000research.183812.r402549

**Published:** 2025-11-03

**Authors:** Maureen Adoyo

**Affiliations:** 1 Information Science, Health Records and Systems, Rongo University (Ringgold ID: 390296), Rongo, Migori County, Kenya; 2 Kenya Medical Research Institute, Nairobi, Nairobi County, Kenya

The paper is adequate in both form and content. The logical flow and documentation of processes are well executed and provide valuable insights that are worth sharing for adoption by countries facing similar HIS challenges. However, there are a few areas that require further elaboration and improvement to enhance clarity and practical applicability

 Regarding the VSOT framework and other approaches mentioned, please clarify whether they were developed during the implementation of the program or adapted from an existing source. If it is an original innovation, consider providing a visual presentation to facilitate adoption by other programs. If not, please acknowledge the source appropriately.

**Methodology**

  It is important to clarify how the number of 17 delegates for participation in the I-LEAD programme was determined. Was this primarily influenced by **a**vailable resources, or were there other criteria e.g., expertise, institutional representation, or regional balance, that guided the decision? Additionally, provide details on the distribution of these delegates among the representative government bodies and agencies.

**Results**

 Check the results section. The sentence *“A detailed roadmap outlining the various tasks to be undertaken across the streamlined activities presented in Table 1 was created and is provided in Appendix 1”* requires revision. Since the process has already been undertaken, I suggest using a reporting verb.

**Discussion**

 I appreciate the flow-chart figure providing an overview of the Nigeria I-LEAD experience across three phases—preparatory activities, the I-LEAD main event, and post-I-LEAD activities. However, the *multiple consultative meetings and discussion sessions* mentioned in the discussion still need further elaboration. It is important to specify the number of these sessions and describe what each session achieved. Please also clarify whether the sessions were designed to address separate objectives or whether each session informed the next convening. This additional information should also be incorporated into the methodology section to guide countries that may wish to replicate the i-LEAD approach to strengthening HIS.

**Conclusion**

 Looking at the conclusion and the stated aim of the *I-LEAD programme aims to empower leaders, managers, and technical experts engaged in public health informatics in envisioning the digital health landscape, formulating effective strategies to realise their vision, and successfully implementing and sustaining these strategies”*—it would strengthen the paper to highlight specific leadership attributes the program developed that are critical for revolutionizing the digital health landscape. Additionally, please indicate any strategies, for example, approaches to sustainable funding developed through this process. Key lessons or highlights in implementing effective and efficient public health informatics or advancing a robust digital health landscape would also provide valuable insights for replication and scale-up.

Is the work clearly and accurately presented and does it cite the current literature?

Yes

If applicable, is the statistical analysis and its interpretation appropriate?

Partly

Are all the source data underlying the results available to ensure full reproducibility?

Yes

Is the study design appropriate and is the work technically sound?

Yes

Are the conclusions drawn adequately supported by the results?

Partly

Are sufficient details of methods and analysis provided to allow replication by others?

Partly

Reviewer Expertise:

Governance, Digital Health, Health Policy, Health System Surveillance for Emerging and Re-emerging Diseases, and Health Information Systems Strengthening.

I confirm that I have read this submission and believe that I have an appropriate level of expertise to confirm that it is of an acceptable scientific standard.

## References

[1] : Intergovernmental Learning Exchange to Advance Data-Driven Decision Making: Experiences from Nigeria. *F1000Research* .2025;14: 10.12688/f1000research.166773.1 10.12688/f1000research.166773.1 41756646 PMC12933044

# Response

**Published:** 2026-02-11

**Authors:** Agbonkhese Oaiya

**Affiliations:** PATH, FCT Abuja, FCT Abuja, Nigeria

Firstly, we sincerely thank you for your thoughtful and encouraging review, and for recognising the manuscript’s overall quality, logical flow, and relevance to countries facing similar Health Information System challenges.

 The following are our responses to the comments raised:

1. The VSOT framework was not developed during the Nigerian I-LEAD implementation. Rather, it was adopted from established literature and operationalised in practice through the combined use of the I-LEAD and GEEKS programmes. We have clarified this distinction in the revised manuscript.
2. The selection of the delegates was based on a balance between the scope of the HIV/AIDS programme in Nigeria and institutions involved in HIS governance, planning and implementation. We have expanded this explanation in the methods section to improve transparency.
3. These consultative sessions in the pre-I-LEAD phase were regular meetings to ensure alignment with agreed objectives and to track progress. We have updated the manuscript to include this description.

We have carefully revised the manuscript in line with the feedback provided.

# Reviewer report: Reviewer response for version 1

**DOI:** 10.5256/f1000research.183812.r409713

**Published:** 2025-09-05

**Authors:** Rok Hrzic

**Affiliations:** 1 Maastricht University, Maastricht, The Netherlands

The authors present a fascinating case study of workforce development in digital health in Nigeria. The manuscript contributes to the emerging literature on best practices in empowering the health and public health workforce to actively engage with the processes of digital transformation in health systems. However, in its current shape, the report lacks essential methodological detail. Below, I offer some reflections that may help the authors strengthen the manuscript.

 1. Clarify data collection and analysis steps

 The manuscript reports on preparatory activities and the outcomes of participation in the I-LEAD program. However, the general analytical approach used to generate these reflections is unclear. How were the participants of the programme selected? Were the participants of the programme interviewed or surveyed to prepare this report? How precisely was the ISHO assessment conducted (by the participants)? What data were collected? How precisely did the various discussion sessions and activities shape the conclusions of the ISHO assessment into the redefined HIS challenges? Answering these questions is a prerequisite for this manuscript contributing to the scientific literature.

 2. Engage with existing literature on workforce development in DH

 The authors focus on the present case study and the Nigerian context. However, it would be helpful for them to more actively engage with similar published case studies and emerging international guidance from WHO and other institutions regarding workforce development in digital health. This will better position the case study in the international literature and highlight its contribution to our knowledge.

 3. Reflect on generalisability and research contribution

 These essential elements are currently absent from the discussion. I invite the authors to expand the discussion by adding them.

 4. Provide more detail on I-LEAD

 I suggest that the authors provide more information on the I-LEAD programme, specifically its origins, development, implementation, and eventual localisation. This will support cross-country learning from this case study and provide readers with much-needed context.

Is the work clearly and accurately presented and does it cite the current literature?

Partly

If applicable, is the statistical analysis and its interpretation appropriate?

Not applicable

Are all the source data underlying the results available to ensure full reproducibility?

No

Is the study design appropriate and is the work technically sound?

Partly

Are the conclusions drawn adequately supported by the results?

Partly

Are sufficient details of methods and analysis provided to allow replication by others?

No

Reviewer Expertise:

comparative health (policy) research, workforce development

I confirm that I have read this submission and believe that I have an appropriate level of expertise to state that I do not consider it to be of an acceptable scientific standard, for reasons outlined above.

# Response

**Published:** 2026-02-11

**Authors:** Agbonkhese Oaiya

**Affiliations:** PATH, FCT Abuja, FCT Abuja, Nigeria

Firstly, we sincerely thank you for your feedback and comments. We have strengthened the manuscript by providing more details per the feedback received. 

 Secondly, the following are our responses to the comments raised:

1. The participants of the programmes were purposefully selected, which was based on the scope of the HIV/AIDS programme in Nigeria. This encompassed representatives from institutions involved in national health information systems’ governance, planning and implementation. The selected participants who are decision-makers provided a balance between the government ministries and agencies overseeing the HIV/AIDS programme and HIS (and informatics), as well as partners supporting this ecosystem in Nigeria.
2. No interview was conducted in selecting these public health informatics leaders across these government institutions overseeing the HIV/AIDS programme in Nigeria.
3. On the ISHO assessment, the expedited above-site ISHO assessment was conducted by the selected and participating delegates using a standardised tool.
4. We have strengthened the methods section of the manuscript, explaining how regular, structured consultative discussions were used to synthesise assessment findings, validate priorities, and refine HIS gaps.
5. We recognise the need to engage in similar case studies, ensuring the manuscript is better positioned in the international literature. However, we also recognise the challenges within the Nigerian HIS ecosystem, especially within the HIV/AIDS programme in meeting both a combined vision of HIV/AIDS epidemic control and the Nigerian government’s goal of a no-AIDS nation. As such, we presented a case of capacity-building for government leadership, linked with embedding realistic challenges and embedding solutions towards what the country is currently facing.
6. We shared and referenced all source data using FigShare, and these are available at: https://doi.org/10.6084/m9.figshare.29345027

We have carefully revised the manuscript in line with the feedback provided.
